# The Delay of Recanalisation of Acutely Thrombosed Dialysis Arteriovenous Access Until the Next Workday has No Negative Impact on Clinical Outcome

**DOI:** 10.1007/s00270-024-03897-5

**Published:** 2024-11-18

**Authors:** Konstantin Hellwig, Stefan Zicha, Christoph Kopp, Ulrich Rother, Nikolaos Papatheodorou, Michael Uder, Axel Schmid

**Affiliations:** 1 Institute of Radiology, University Hospital of Erlangen, Friedrich-Alexander University Erlangen-Nuremberg, Maximiliansplatz 3, 91054 Erlangen, Germany; 2 Department of Nephrology and Hypertension, Friedrich-Alexander University Erlangen-Nuremberg, 91054 Erlangen, Germany; 3 Department of Vascular Surgery, University Hospital Erlangen, Friedrich-Alexander University Erlangen-Nuremberg, 91054 Erlangen, Germany

**Keywords:** Hemodialysis, Arteriovenous fistula, Thrombosis, Recanalization, Endovascular

## Abstract

**Purpose:**

The necessity of providing endovascular recanalization of acutely thrombosed arteriovenous access (AV access) during weekend is questionable, since hemodialysis can alternatively be achieved by temporarily placed non-tunneled central venous catheters (CVC). Interventional therapy of acutely thrombosed AV access is provided only on weekdays in the study center. This study aimed to compare outcomes in patients admitted on weekdays and on the weekend.

**Methods:**

A total of 355 endovascular procedures for thrombosed AV access performed in a single tertiary center from 2007 to 2017 were retrospectively analyzed for technical and clinical success, complications, rate of CVC and length of hospitalization. Technical success was defined as adequate blood flow with less than 30% residual stenosis, clinical success was defined as at least one successful hemodialysis after recanalization. There were two groups: patients who had to wait at least 2 days for recanalization due to admission at the weekend (*n* = 59, at-the-weekend group, ATW group) and patients receiving therapy no later than the day after admission (*n* = 296, on a working day group, OAW group).

**Results:**

The technical/clinical success rate was 96.6%/88.1% in the ATW and 89.1%/84.6% in the OAW group (*p* > .05). Complications did not differ among groups (*p* > .05). Despite a higher rate of CVC, no attributed additional adverse events or complications were observed in the ATW group (*p* > .05).

**Conclusion:**

Despite a longer time until treatment and a higher rate of short-term CVC, it seems to be justified to provide recanalization of dialysis shunts only during weekdays.

**Graphical Abstract:**

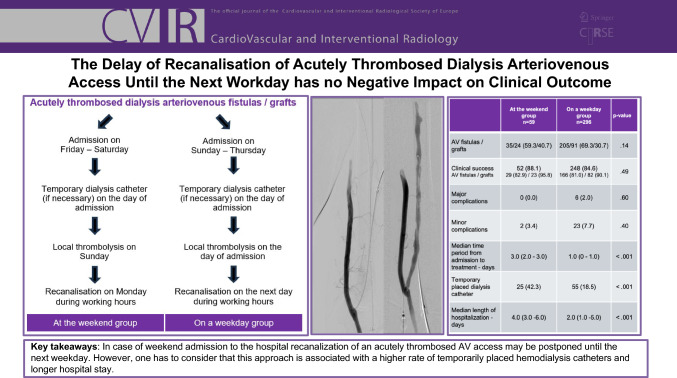

## Introduction

Acute thrombosis of arteriovenous fistula (AVF)/arteriovenous graft (AVG) is a life-threatening complication of vascular access and requires treatment in a timely manner. Recanalization of the AV access can be either by surgical or interventional thrombectomy. While, surgical thrombectomy was the standard procedure up until the 1990s, interventional techniques for recanalization of thrombosed AV access have become the prevailing method of recanalization [1, 2]. Although endovascular treatment is associated with shorter hospital stays and a lower rate in wound complications [3], interventional percutaneous recanalization remains a time-consuming and demanding procedure which requires both experienced physicians and technicians. In particular, outside of normal working hours there may be a lack of specialized teams in many centers. Multiple studies have already described the so called “Weekend-Effect” for interventional and surgical procedures. Procedures performed during weekends show poorer outcome compared to the same procedure performed during weekdays [4–7]. Taking this into consideration, the medical necessity of immediate recanalization of an acutely thrombosed AV access on weekends is questionable since hemodialysis if necessary can be facilitated via temporarily placed non-tunneled CVCs. The possible complication rate of temporarily placed catheters is low, but patients might not want to get an additional catheter.

In the study center endovascular treatment of acutely thrombosed fistulas and grafts is provided only on working days using the “lyse and wait” technique with time extended local thrombolysis, where necessary. The aim of this study was to retrospectively compare the complications and clinical outcomes of endovascular dialysis shunt recanalization between patients treated promptly (within 1 day after admission) and those who had to wait at least two days for treatment due to weekend admission.

## Materials and Methods

### Study Design

All patients who received interventional recanalization of acutely thrombosed hemodialysis access in the study center from September 25th 2007 to October 2nd 2017 were included in this study. Exclusion criteria were central vein occlusion and partially thrombosed AVF/AVG. Data of all procedures was collected from the patient files including demographic data, clinical assessment, technical and clinical success, rate of complications, rate of temporarily placed dialysis catheters, additional adverse events between admission until treatment and length of hospitalization. The study was conducted in accordance with the Declaration of Helsinki and further approved by the local ethics committee (166_20 Bc). For this type of study formal consent is not required.

In most interventions (*n* = 263, 74.0%) recanalization was performed after local thrombolysis. The use of local thrombolysis was at the discretion of the interventionalist depending on the amount of thrombus and the length of occlusion. Patients were grouped according to the time of admission: at-the-weekend group (ATW group) and on-a-working day group (OAW group). The ATW group were procedures with admission to hospital on Friday or Saturday in which recanalization was performed on the following Monday after time-extended local thrombolysis on Sunday if required. The OAW group consisted of patients admitted from Sunday to Thursday, treated by thrombolysis if necessary on the day of admission followed by recanalization the next day. A working day is considered Monday until Friday from 7:30 am to 5:30 pm. The decision for CVC placement was made by the nephrologist on call, based on potassium level and patient condition.

### Procedure

Recanalization of the acutely thrombosed AV access was performed as described previously by Regus et al. [8]: If local thrombolysis was indicated, 2–5 mg of recombinant tissue plasminogen activator (rtPA) with 2000–3000 International Units (IU) of Heparin were administered in a single or a split bolus into the thrombosed access segment via one or two peripheral venous catheters prior to intervention. In AVF the peripheral venous catheter remained until successful recanalization in order to reduce the risk of bleeding in case of spontaneous recanalization during the waiting period as a result of local thrombolysis. In AVG the venflon was removed in order to prevent shunt infection. A time period between lysis and endovascular recanalization shorter than three hours was considered short term thrombolysis (STT), local lysis therapy exceeding three hours was regarded as long term thrombolysis (LTT). There was no significant difference in the amount of applied rtPA/Heparin when comparing STT and LTT. The interventional procedure was performed either via one or two access points depending on the location of thrombus. It consisted of balloon angioplasty of high grade stenoses, mechanical fragmentation and mobilization of thrombotic material with balloons, 4 or 5F RIM catheters (Tempo™, Cordis) or percutaneous mechanical thrombectomy devices and manual aspiration thrombectomy, where possible. Sufficient blood flow was documented at the end of every procedure.

Technical success was defined as adequate blood flow with a residual stenosis less than 30%, clinical success was considered as at least one successful dialysis following the intervention.

Complications were rated according the “The CIRSE Classification System” of the Cardiovascular and Interventional Radiological Society of Europe (CIRSE) into minor (grade 1) and major complications (grade 2–6) [9]. Additionally, serum potassium levels at admission, rate of temporarily placed dialysis catheters, and rate of additional clinical adverse events during the time period between admission and treatment and length of hospitalization were evaluated. Patients with an acute thrombosis of AV access during a hospital due to other medical reasons were excluded from the analysis in regard to hospitalization length.

### Statistics

Statistical analyses were performed with IBM SPSS Statistics for Windows, version 26 (IBM Corp., Armonk, NY, USA). The level of significance was defined as *p* < 0.05. Testing was performed with the Mann–Whitney *U* test for independent samples, Pearson’s chi-squared test and Student's t-test. Shapiro–Wilk test was utilized to check for normal distribution.

## Results

In this observational single-center study 355 procedures of endovascularly treated acutely thrombosed dialysis fistulas/grafts (fistulas *n* = 240, grafts *n* = 115) in 188 consecutive patients were retrospectively analyzed. 118 patients were male (62.8%) and 70 were female (37.2%). Mean age was 65.6 years (standard deviation 15.8 years).

From the 355 interventions 59 procedures (16.6%) in 43 patients were counted among the ATW group and 296 procedures (83.4%) in 171 patients among the OAW group (Table 1). In the same time period, a total of 26 surgical thrombectomies were performed in patients suitable for the ATW group. Ten surgical treated patients would have also been suitable for interventional recanalization. In the remaining 16 patients additional surgical procedures such as revision of the anastomosis or resection of an aneurysm had to be conducted. In the ATW group local thrombolysis was used in a significantly higher number of interventions compared to the OAW group [ATW 50/59 (84.7%)–OAW group 213/296 (72.0%); *p* = 0.041]. In the majority of both groups thrombolysis was conducted on the day prior to recanalization as time extended local lysis. The median amount of rTPA and heparin used for thrombolysis did not differ between the groups (see Table 1).

**Table 1:** Patient characteristics and clinical data for both on a working day group and at the weekend group, level of significance was defined *p* < .05; data are presented as *n* (%), mean ± standard deviation or median (interquartile range), IU = international units, rtPA = recombinant tissue plasminogen activator

	At the weekend group *n* = 59	On a weekday group *n* = 296	*p* value
Age—years	67.1 ± 16.4	64.7 ± 15.7	.074
Male	32 (54)	177 (60)	.43
AV fistulas/grafts	35/24 (59.3/40.7)	205/91 (69.3/30.7)	.14
Technical success	57 (96.6)	264 (89.1)	.077
AV fistulas/grafts	33 (94.3)/24 (100)	178 (86.8)/86 (94.5)	
Clinical success	52 (88.1)	248 (84.6)	.49
AV fistulas/grafts	29 (82.9)/23 (95.8)	166 (81.0)/82 (90.1)	
Interventions with local lysis therapy	50 (84.7)	213 (72)	.041
Time extended lysis / Short term lysis (< 3 h)	48 (81.4)/2 (3.4)	204 (68.9)/9 (3.0)	
Median amount of local rtPA—mg/local heparin—IU	3.0 (2.0–5.0)/	3.0 (2.0–4.0)/	.97/.15
	2000 (1000–2500)	2000 (2000–2500)	
Major complications	0 (0.0)	6 (2.0)	.60
Minor complications	2 (3.4)	23 (7.7)	.40
Median time period from admission to treatment—days	3.0 (2.0–3.0)	1.0 (0–1.0)	< .001
Median serum potassium level at admission—mmol/l	5.8 (4.9–6.6)	5.7 (5.1–6.5)	.64
Temporary placed dialysis catheter	25 (42.3)	55 (18.5)	< .001
Median length of hospitalization—days	4.0 (3.0–6.0)	2.0 (1.0–5.0)	< .001
30 day mortality	2 (3.4)	17 (5.7)	.75

There was no significant difference in the rate of technical success between the two groups [ATW group 57/59 (96.6%)–OAW group 264/296 (89.1%); n.s.] and clinical success [ATW group 52/59 (88.1%)–OAW group 248/296 (84.6%); n.s.]. No major complications were observed in the ATW group, while six major complications occurred in the OAW group (6 CIRSE category 3). Said complications consisted of three shunt vein hemorrhagic events requiring blood transfusion, two shunt infections which required antibiotic therapy and one case axillary artery thrombosis necessitating surgical thrombectomy. In this last case of axillary artery thrombosis there was no apparent association with the intervention. The rate of minor complications was 2/59 (3.4%) in the ATW group and 23/296 (7.8%) in the OAW group. There was no significant differences in major and minor complication rates.

The median serum potassium level at admission was 5.8 mmol/l (IQR 4.9–6.6) in the ATW group and 5.7 mmol/l (IQR 5.1–6.5 mmol/l) in the OAW group (n.s.). Medical therapy with sodium polystyrene sulfonate was given in 30/59 patients (50.8%) in the ATW group and in 100/296 patients (33.7%) in the OAW group (*p* = 0.01). Significantly more temporary dialysis catheters were placed in the ATW group [25/59 procedures (42.3%)] compared to the OAW group [55/296 procedures (18.5%); *p* < 0.001]. Decision for CVC were made at a lower mean potassium level in the ATW group (6.3 mmol/l) compared to the OAW group (6.97 mmol/l; *p* = 0.003). No complication resulting from CVC was registered in the ATW group, one complication (catheter associated infection) was observed in the OAW group (n.s.).

The median time period from admission to recanalization (3.0d in the ATW group–1.0d in the OAW group; *p* < 0.001) and median length of hospitalization (4.0 days in the ATW group–2.0 days in the OAW group; *p* < 0.001) were significantly longer in the ATW group. No adverse events occurred in either group from admission to final therapy. Mortality 30 days after the intervention did not significantly differ between the ATW group (*n* = 2, 3.4%) and the OAW group (*n* = 17, 5.7%; n.s.), none of the deaths was related with the recanalization of the thrombosed AVF.

## Discussion

Acute thrombotic occlusion of an AV access represents a critical event for dialysis patients which requires timely treatment. Medical institutions have to determine how to deal with this problem in case of weekend admission. Patients and their referring nephrologists often expect immediate recanalization of a thrombosed hemodialysis shunt. However, this requires the interventional radiology or vascular surgery department to provide constant stand-by of medical professionals skilled in the treatment of an occluded vascular access. In particular, the endovascular approach to recanalization poses a challenging and difficult procedure requiring a high level of experience from interventional radiologist and the availability of specially trained radiological technologists. In addition, there might be other urgent interventions such as acute bleeding or acute limb ischemia decreasing the availability of the interventionalist team. Moreover, it is questionable if there is a medical need for early recanalization of the AVF on weekends due to the fact that in many patients potassium levels can be controlled nutritionally and pharmacologically for a certain amount of time (1–2 days). Additionally, hemodialysis if required can be achieved via temporarily placed dialysis catheters. Although current guidelines recommend “thrombectomy should be performed in a timely fashion relative to the event, particularly for AVFs” or “treatment needs to be started as soon as possible” it still remains unclear what is meant by the mentioned time [10, 11]. It is still open, whether a delay has an influence on the outcome of these recanalization procedures.

The data of this study suggests, that postponing timely recanalization on weekends does not pose additional medical risk to hemodialysis patients. In the ATW group neither adverse events during the period from admission to treatment nor complications associated with dialysis catheter placement occurred. Even if the rate of short term CVC was significantly higher in the ATW group the potassium levels in the majority of patients were either uncritical or able to be managed by pharmacological treatment until final treatment after the weekend. Furthermore, technical and clinical success rate appeared to be higher in the ATW group compared to the OAW group. This could possibly be explained by a more frequent use of local thrombolysis in the ATW group. Time extended local lysis has been shown to lead to shorter intervention times and a lower rate of complications [8]. Overall, the technical and clinical success rate in our cohort equals those published on endovascular treatment of thrombotic occlusion of the vascular access varying between 59 and 99% (see Table 2) [12–32].

**Table 2:** Review of the literature on recanalization of thrombosed arteriovenous fistulas, * in 28 procedures (9.2%) pharmacological therapy alone was administered; n.a. = not available

Authors	Year	*N*	Success rate	Major/minor complications
Poulain et al.	1991	64	59% (technical)	12.5%/n.a
Sands et al.	1994	71	91% (technical)	4.2%/9.9%
Haage et al.	2000	81	88.9% (technical)	1.2%/3.7%
Vogel et al.	2001	40	95% (technical)	7.5%/10.0%
Sofocelous et al.	2002	27	94% (technical)	5.9%/5.9%
Cooper et al.	2003	17	94% (technical & clinical)	0% / 0%
Schon et al.	2003	25	82% (technical)	8.0%/n.a
Vashchenko et al.	2010	427	99% (technical)	4.2%/n.a
Choi et al.	2012	82	95% (technical & clinical)	0%/10.0%
Umanath et al.	2012	321	73.8% (technical)	2.5%/3.1%
Lee et al.	2014	75	89.3% (clinical)	0%/6.6%
Nassar et al.	2015	520	91.1% (clinical)	0.8%/0.5%
Nikam et al.	2015	410	93% (technical)	0.5%/5.5%
Regus et al.	2018	152	89.5% (clinical)	3.3%/n.a
Tan et al.	2019	122	98.4% (technical) 93.4% (clinical)	0.8%/10.7%
So et al.	2019	73	93% (technical) 85% (clinical)	0.0%/6.8%
Yilmazsoy et al.	2019	304*	98.7% (technical)	1.3%/0.6%
Leo et al.	2020	130	93.8% (technical) 95.2%/84.2% for immature fistulas (clinical)	3.0%/2.3%
Tan et al.	2020	294	96.3% (technical) 93.2% (clinical)	0.7%/9.9%
Lundström et al.	2022	368	91% (clinical)	n.a
Deogaonkar et al.	2023	328	87.8% (technical) 75.9% (clinical)	5.2%/13.1%
Hellwig et al.	2023	355	90.4% (technical) 84.5% (clinical)	1.7%/7.0%

This study has several limitations. First of all, the data is not suitable to directly compare results of endovascular therapy on weekends with those on regular working days, since all patients in this cohort were eventually treated on weekdays.

Beyond that, the study data was gathered retrospectively which could potentially result in an underestimation of adverse events due to a possible incomplete documentation in the patient charts or lacking further evaluation by a systematic follow up. In particular, the incidence of late central venous stenosis caused by the placement of CVC remains beyond the scope of this study. The number of placed central catheters and the total sum of catheter days are known risk factors for the occurrence of late central venous stenosis [33–35]. However, it remains unclear as to how far the rate of central venous stenosis is affected by ultra-short-termed non-tunneled CVCs for two days or less. Studies on the impact of short-term hemodialysis catheters on central veins included only patients with at least seven catheter days [36, 37].

Furthermore, the indication for CVC placement was not standardized and was based on the decision of the nephrologist on call. Therefore, the rate of CVC placement could be biased in either group. Finally, even if the total number of procedures in this study is comparatively high, the subgroup of patients admitted at the weekend represent only a minor proportion and therefore, final conclusions on the optimal treatment approach in this group of patients have to be viewed with caution.

## Conclusion

In case of weekend admission to the hospital recanalization of an acutely thrombosed AV access may be postponed until the next weekday. However, one has to consider that this approach is associated with a higher rate of temporarily placed hemodialysis catheters and longer hospital stay.
